# On the gender–science stereotypes held by scientists: explicit accord with gender-ratios, implicit accord with scientific identity

**DOI:** 10.3389/fpsyg.2015.00415

**Published:** 2015-04-27

**Authors:** Frederick L. Smyth, Brian A. Nosek

**Affiliations:** ^1^Department of Psychology, College and Graduate School of Arts and SciencesCharlottesville, VA, USA; ^2^The Center for Open Science, University of VirginiaCharlottesville, VA, USA

**Keywords:** diversity, gender, science education, science workforce, stereotypes

## Abstract

Women's representation in science has changed substantially, but unevenly, over the past 40 years. In health and biological sciences, for example, women's representation among U.S. scientists is now on par with or greater than men's, while in physical sciences and engineering they remain a clear minority. We investigated whether variation in proportions of women in scientific disciplines is related to differing levels of male-favoring explicit or implicit stereotypes held by students and scientists in each discipline. We hypothesized that science-is-male stereotypes would be weaker in disciplines where women are better represented. This prediction was tested with a sample of 176,935 college-educated participants (70% female), including thousands of engineers, physicians, and scientists. The prediction was supported for the explicit stereotype, but not for the implicit stereotype. Implicit stereotype strength did not correspond with disciplines' gender ratios, but, rather, correlated with two indicators of disciplines' scientific intensity, positively for men and negatively for women. From age 18 on, women who majored or worked in disciplines perceived as more scientific had substantially weaker science-is-male stereotypes than did men in the same disciplines, with gender differences larger than 0.8 standard deviations in the most scientifically-perceived disciplines. Further, particularly for women, differences in the strength of implicit stereotypes across scientific disciplines corresponded with the strength of scientific values held by women in the disciplines. These results are discussed in the context of dual process theory of mental operation and balanced identity theory. The findings point to the need for longitudinal study of the factors' affecting development of adults' and, especially, children's implicit gender stereotypes and scientific identity.

## Introduction

In 1966 just 7% of undergraduate women took their bachelor's degrees in STEM (Science, Technology, Engineering, Math, excluding health and social science; National Science Foundation, National Center for Science and Engineering Statistics, 2011a, Table 9). More than four decades later, despite passage in 1972 of Title IX of the Civil Rights Act and an ensuing great expansion of higher education opportunities for women, the figure moved only to 10% (National Science Foundation, National Center for Science and Engineering Statistics, 2011a, data for 2008). This is slightly down from the high water mark of 12%, first reached during the mid-1980s and achieved again in 2000 and 2003. Meanwhile, men's likelihood of majoring in STEM disciplines decreased, from 29% in 1966 to 23% in 2008 (National Science Foundation, National Center for Science and Engineering Statistics, 2011a, Table 7). These trends—slow, halting progress into STEM for women and declining interest for men—may explain why leaders in STEM fields are concerned with recruitment and retention of everyone, regardless of sex, but also draw attention to the persisting sex difference in pursuit of STEM-related careers. Even with men's sagging interest, in 2008 they were still more than twice as likely as women to pursue and earn an undergraduate STEM degree.

Eliminating the apparent ceiling on women's STEM interest has long been a national priority, its causes and possible remedies the focus of extensive research and debate (e.g., Gallagher and Kaufman, 2005; Summers, 2005; Ceci and Williams, 2007, 2010, 2011; Halpern et al., 2007; National Academies of Science, 2007). Increasing attention has been paid to the variation in women's representation across different STEM domains (e.g., greater in biology than in engineering; National Science Foundation, National Center for Science and Engineering Statistics, 2011a) as a clue to understanding the causes of their underrepresentation (Su et al., 2009; Robertson et al., 2010; Cheryan, 2012).

## Variation in STEM gender ratios

### Undergraduate degrees

**Figure 1:**
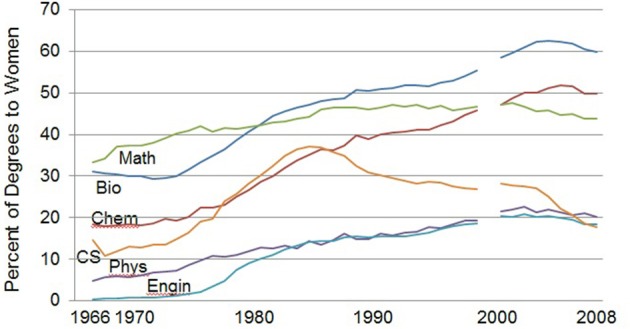
**Percentage of women earning bachelors degrees awarded in STEM, 1966–2008 (NSF, 2011a; Tables 5, 7, 9, 28, 33, 34, 37, 38, and 46)**. Bio, Biology; CS, computer sciences; Phys, Physics; Engin, Engineering.

Beneath the overall sex difference in STEM pursuit there is wide variation in STEM gender ratios across disciplines. Figure 1 plots the percentage of women earning the bachelor's degrees awarded in various major STEM fields from 1966 to 2008. By 2008 women earned between 44 and 60% of the degrees in biology, chemistry and mathematics, but only about 20% in physics, engineering, and computer science (National Science Foundation, National Center for Science and Engineering Statistics, 2011a).

### Occupations

Variation in women's STEM representation is also apparent among practicing scientists. In 2006, among employed U.S. scientists (defined by NSF as anyone in a STEM field with at least a bachelor's degree; National Science Foundation, 2011b) women constituted nearly two-thirds (65%) of those in health sciences, 50% of those in biological sciences, 27% in physical sciences (including 33% in chemistry, 21% in earth sciences, and 16% in physics/astronomy), and 13% in engineering. Women now constitute roughly half of all new physicians (Association of American Medical Colleges, 2013), but among employed PhD-level scientists, women comprise just 16% in the physical sciences and 9% in engineering (National Science Foundation, 2011b). Thus, in the professional scientific ranks, biological and health sciences are characterized by relatively high female-male ratios, at 1:1 or higher, while physical sciences and engineering are characterized by low female-male ratios, at 0.33:1 or lower.

## The influence of stereotypes

Recent studies indicate that variability in women's engagement across STEM fields reflects patterns of early-developing childhood interests, and that these interests may be influenced by stereotypes and by inadequate information about the nature of opportunities in different scientific domains (Ceci and Williams, 2011; Cheryan, 2012; Eccles, 2007; Kaminski and Geisler, 2012). Although stereotypes about gender and STEM (e.g., more naturally the domain of boys and men) are now usually explicitly disavowed as a rationale for choosing courses to take, majors to enter, or persons to hire, evidence suggests that they nevertheless affect perceptions, performance and decisions, primarily without intention or awareness (e.g., Crowley et al., 2001; Moss-Racusin et al., 2012; Nosek et al., 2012; Galdi et al., 2014). Stereotypes, generally defined as associations of an attribute with members of a group, can operate at an “explicit” level, i.e., conscious perceptions of, or beliefs about, group-attribute covariation, and also at an “implicit” level, as automatic, possibly unwanted, group-attribute associations that operate outside of conscious awareness (Greenwald and Banaji, 1995; Lee et al., 1995; Gawronski and Bodenhausen, 2006; Nosek et al., 2012).

A naturalistic observational study of families at science museums seems to illustrate the independence of explicit and implicit gender–science stereotypes. Crowley et al. (2001) found that parents who brought their children to science museum exhibits spontaneously offered more explanations of phenomena to their sons than to their daughters. Here were parents that, ostensibly, were working to expose both their girls and boys to science, yet, unknowingly, were engaging more, teaching more, with the boys. If asked, these parents would doubtless say (and believe)—explicitly—that they are equally committed to the best possible science education for their child of either sex; that's why they were visiting the science museum! But Crowley et al.'s observations of many families belie differential treatment according to sex of the children, an implicit bias impossible for any of these parents to observe on their own. Such unconscious sex-differentiated patterns in adults' interaction with children in the science domain are the sort that Galdi et al. (2014) speculated may spur the early development of girls' implicit math–gender stereotypes, which they found operating for six-year-olds prior to the emergence of explicit stereotyping. Galdi et al. (2014) experimentally exposed six-year-old boys and girls to either stereotype-congruent or incongruent images of children and math accomplishment and observed corresponding influence on the girls', but not the boys', implicit math(language)–gender stereotypes. The induced implicit stereotyping differences, in turn, were found to mediate stereotype-consistent effects on the girls' math performance, while there were no effects of explicit endorsement of math–gender stereotypes. If parents' and teachers' unconscious behaviors systematically suggest that certain STEM disciplines are more fitting for one sex than the other, the effect on children's implicit stereotypes, accumulating from a very young age, may differentially influence interest, accomplishment and persistence in particular sciences.

## Relations between gender ratios and stereotypes

Our data allowed investigation of whether variation in female representation across scientific disciplines is associated with differences in the strength of gender–science stereotypes, explicit and implicit, held by men and women in these fields. Current theory and evidence suggests that both explicit and implicit gender–science stereotypes should change as conditions in local environments change, including gender ratios. In perhaps the most relevant work supporting this idea, Miller et al. (in press), using the same stereotype measures we will analyze, found that average explicit and implicit stereotypes across 66 countries correlated negatively with countries' female proportion of college science majors; that is, higher proportions of women in collegiate science predicted weaker country-level science-is-male stereotyping.

Gawronski and Bodenhausen (2006), in their associative-propositional evaluation (APE) model, argue that explicit evaluations, such as stereotypes, ultimately depend on weighing the truth and importance of propositions that come to mind, e.g., “When I look around in my physics class I see mostly men.” or “I'm a woman doing very well in physics.” When answering a question about degree of association between science and gender, if women in physics take stock of gender ratios, they will see, on average, fewer women than will be seen by women in biology. Thus, other factors being equal, physics women should explicitly report a stronger science-is-male association than should biology women. This is consistent with Eagly and colleagues' social role theory (Eagly and Steffens, 1984; Eagly et al., 2000) which posits that varying distributions of men and women in certain activities and occupations drive explicit stereotyping and promote a cycle of corresponding skill and interest development. Consistent with such a cycle, Inzlicht and Ben-Zeev (2000), studying a sample of students from a highly selective private university (though not from any particular academic major) experimentally demonstrated a connection between gender ratios, stereotypes and academic performance. They found that women's math, but not verbal, test scores suffered as a function of increased proportion of men in the immediate enviroment. Diekman and Eagly (2000) demonstrated that explicit stereotypes are responsive to changes in women's representation; if gender distributions change, explicit stereotypes follow suit.

Implicit stereotyping, too, should vary with gender ratios. Ratliff and Nosek (2010) demonstrated that implicit associations quickly form in accord with environmental stimuli. Gawronski and Bodenhausen's (2006) APE model specifies that change in implicit evaluation will follow from either a changed structure of mental associations (actual strengthening of the associative link between a group and an attribute) or from the differential activation of existing structures (e.g., science–male associations may be more likely to be activated if one is routinely surrounded by men in a scientific context). Thus, for both men and women studying or working in scientific environments with higher male-to-female ratios, we can expect either route to result, on average, in stronger implicit science-is-male stereotyping. Miller et al. (in press), found that the negative country-level relation between female proportion of science majors and implicit science-is-male stereotyping was stronger for participants with college experience than for those without, suggesting that greater associative exposure to particular collegiate gender–science ratios may be the difference.

Results of studies of *change* of implicit stereotypes as a function of gender representation in the environment, however, are mixed. Stout et al. (2011) found no change in the math–gender stereotype evidenced by female calculus students as a function of the sex of their professor, even though strong positive change was observed for these women's implicit math attitude and identity. Consequently, Dasgupta (2011) argues that implicit STEM–gender *stereotypes* are rather intractable, but that their effects can be neutralized to the extent that implicit STEM *identity* is strong, and that the latter strengthens with increased exposure to female faculty and competent STEM peers. Smyth and colleagues (Martin et al., 2013; Smyth unpublished data), studying the math–gender stereotypes of students in university differential equations courses with female professors, found that implicit stereotype change depended on the sex of the student. Women's stereotypes, relatively weak to begin with, did not change, but men evidenced statistically significant weakening of their initially strong stereotype. Perhaps the strongest evidence for implicit stereotype change as a function of gender ratios in the local environment comes from change in a leadership-is-male stereotype (Dasgupta and Asgari, 2004). The strength of women's stereotypes changed across the first year of college depending on their degree of contact with female faculty, weakening with greater contact. Their results imply, Dasgupta and Asgari concluded, that increased female representation in local environments in previously male-dominated fields can, even in the short space of a year, “… have a powerful impact on stereotype change” (p. 654).

Greenwald and colleagues' balanced identity theory (BIT) of implicit social cognition (Greenwald et al., 2002) is grounded in principles of cognitive consistency and balanced identity (Heider, 1958). BIT anticipates that change in any one of these three sets of associations—group identity (e.g., self–female), attribute identity (e.g., self–math), or stereotype (e.g., math–male)—will induce balancing change in at least one of the others. Thus, if women's self-identification strengthens with the male-stereotyped field of math, as found by Stout et al. (2011), BIT predicts weakening of either their implicit female gender identity or their implicit math-is-male stereotype, or both, to maintain congruence or cognitive balance among the associations. If girls' and women's science identity is strengthened by increased opportunity to interact with female peers and mentors in scientific endeavors (as suggested by Dasgupta, 2011), then according to BIT we should find weaker science-is-male implicit stereotypes among women in high female-male ratio science fields than among those in low female-male ratio science fields. In other words, if their self–science associations strengthen, and their self–female associations hold constant, then their counter-stereotypical female–science associations will strengthen—and their stereotypical male–science associations will weaken.

There is abundant evidence that implicit STEM–gender stereotypes are not monolithic, but vary predictably with interest, persistence, and performance in math and science (Nosek et al., 2002a; Kiefer and Sekaquaptewa, 2007; Nosek and Smyth, 2011; Lane et al., 2012). As predicted by BIT, men and women who identify with science differ substantially in the strength of their implicit gender stereotypes about science and math (Nosek et al., 2002a; Nosek and Smyth, 2011; Lane et al., 2012). For men, stronger science self-concepts are associated with stronger implicit science-is-male bias, while for women stronger science self-concepts coincide with weaker implicit science-is-male bias. Nosek and Smyth (2011), studying data from other online volunteers, found a trend of weaker implicit math-is-male stereotyping for both men and women who pursued graduate work in STEM compared to those with only undergraduate training (between 0.1 and 0.2 standard deviations weaker). The much larger current data set, which includes more detailed reports of degree-level and specifications of different STEM disciplines, allows testing of “dosage” effects within particular fields. Does prolonged exposure to a particular gender-ratio correlate with stereotype strength differences within given fields? That is, do scientists in low-female fields evidence stronger science-is-male stereotypes, and scientists in high-female fields evidence weaker ones, the longer they practice in that field?

## Hypotheses about variation in the strength of science-is-male stereotyping

1. Stereotype differences between female and male scientists:
  - Implicit: Women who are strongly identified with science will have relatively weak implicit stereotypes, while men who are strongly science-identified will have relatively strong ones. This pattern, already well-established in the literature based on broad classifications like STEM vs. not-STEM, should yield large sex differences in implicit stereotyping among the scientists of our sample. Our data, which includes more detailed distinctions of academic major and profession than collected in other studies of implicit stereotyping in STEM, allows a more fine-grained replication of this well-established pattern.
  - Explicit: The same pattern of sex difference will hold for scientists' explicit stereotyping, weaker for women, stronger for men, if only because self–science propositions that may come to mind will differ (“I'm successful in science and a woman.” vs. “I'm successful in science and a man.”). However, owing to conscious endorsement of egalitarian values and social approbation against stereotyping, we expected the explicit stereotyping gender gap to be smaller than for implicit stereotypes.
2. Stereotype differences as a function of gender ratios in science environments:
  - Implicit: Science-is-male stereotypes will be stronger for both women and men in low-female STEM fields than in high-female fields, though sex differences should remain. For example, women in physics (low-female) should evidence stronger implicit science-is-male stereotyping than women in biology (high-female). The pattern for men in these majors should be similar, even if the means are higher than women's.
  - Explicit stereotypes will also reflect gender ratios (stronger stereotypic associations reported in low-female than in high-female science disciplines). Again, however, group variation on explicit stereotype means should be somewhat constrained by conscious values and motivations to respond without bias.
3. Stereotype differences as a function of “dosage” of exposure to given gender-ratios:
  - Implicit: Prolonged exposure to STEM environments characterized by particular gender ratios will strengthen the corresponding implicit stereotype. That is, prolonged exposure to low-female environments should strengthen science-is-male stereotyping, while prolonged exposure to high-female environments should weaken it. This hypothesis derives from theory and empirical findings concerning the formation of new implicit associations, and some cross-sectional data that are consistent with dosage effects. Nosek and Smyth (2011) found a slight diminution of stereotyping for both men and women reporting graduate study in STEM, compared to those with only undergraduate study, and Miller et al. (in press) found a college vs. no-college effect on stereotyping as a function of collegiate gender ratios in STEM, generally. Neither of these analyses distinguished between types of STEM fields. In the current data, we expect increasing stereotype-strength from age 18 to age 22 among science-declared college students in low-female fields, and a declining trend in high-female fields. Similar patterns should be found across increasing levels of training (e.g., from BS to MA to PhD).
  - Explicit stereotypes, when measured for scientists in a given field with roughly constant gender-ratio, will not be systematically responsive to dosage because the general propositions being consciously weighed may not change very much. That is, whether for an undergraduate woman majoring in physics or a female professor of physics, the propositions to weigh will likely involve, on average (1) the fact of the majority-male field and (2) assessments of personal, or other women's, accomplishments. If noteworthy scientific accomplishments by women come to mind easier for women who have been in the field longer, we might expect a diminution of the explicit stereotype. But if the intractability of the gender-ratio in the field is more salient for these women, their stereotype self-reports might strengthen. To the extent that these different framings are idiosyncratically applied by individuals, systematic change across cohorts would seem unlikely.

### Study overview

We tested these predictions with over 176,000 visitors to a publicly accessible educational website (https://implicit.harvard.edu/) who reported U.S. citizenship, at least some college experience and an academic major. Explicit “science-is-male” stereotyping was defined simply as verbally associating the term “science” more with “male” than with “female,” and implicit stereotyping by performance on an Implicit Association Test, fully described in the methods section. Our data are cross-sectional, so differences across age or level of training can only be considered suggestive of change. A particular strength of our sample is inclusion of thousands of STEM majors, whereas most other research on implicit STEM associations has been conducted with small samples.

A public website, known as *Project Implicit*, was launched in September 1998 with the purpose of heightening public awareness of implicit social cognition, and alerting participants to the possibility that mental associations outside of their awareness or control might differ from their consciously held attitudes (Nosek et al., 2002b, 2007). Visitors to the site may choose from a variety of Implicit Association Tests (IATs; Greenwald et al., 1998) and “Gender–science” has been a long-standing and popular topic (for summary of the topics and data, see Nosek et al., 2007). Though the sample is not representative of a definable population other than that of visitors to the *Project Implicit* site, it reflects greater age and education variation than the samples of college students that characterize much research. Study protocol was reviewed and authorized by the University of Virginia *Institutional Review Board for Social and Behavioral Sciences*.

## Methods

### Participants

Our analyses are of 176,935 *Project Implicit* volunteers from May 2004 through January 2012 who reported U.S. citizenship, their sex, at least some college experience, an academic major, and completed the implicit and explicit academic stereotype measures. Seventy percent of participants were female, and racial identifications, in order of proportion, were White, 81.1%; Black, 5.5%; More than one race, 5.1%; Other or unknown, 3.1%; East Asian, 2.6%; South Asian, 1.5%; American Indian/Alaska Native, 0.6%; Native Hawaiian/Pacific Islander, 0.5%. An Hispanic ethnicity was reported by 6.7%, not-Hispanic by 89.3%, and unknown ethnicity by 4.0%^1^. The median age was 25 (*M* = 29, *SD* = 12, range 17–90), and 59% were older than the typical college age range of 17–22. Fifty-one percent of participants reported some college experience short of a bachelor's degree (most of these were aged 18–22), 30% reported a bachelor's degree as their highest level, and 19% reported a graduate degree.

### Explicit science identity: academic major

Participants could select from the following list of 13 categories of majors to indicate their “Major field of study or that of your highest degree.” Underlined categories were coded as STEM majors in our analyses.

Biological sciences/life sciences
Business
Communications
Computer and information sciences
Education
Engineering, mathematics, or physical sciences/science
technologies
Health professions or related sciences
Humanities/liberal arts
Law or legal studies
Psychology
Social sciences or history
Visual or performing arts
Other

**Figure 2:**
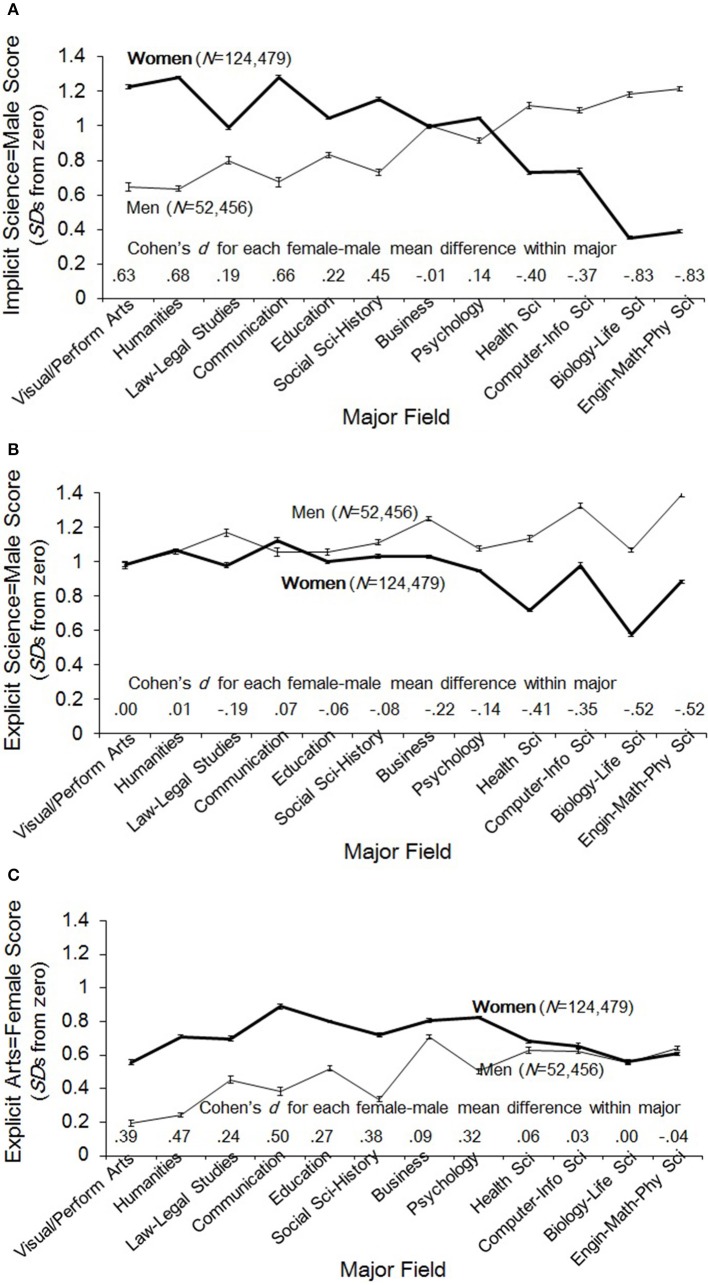
**(A)** Mean implicit science = male IAT score (+/- 1 *se*) by sex and major field. Majors are ordered, left to right, by ratings of science content (method described in Supplementary Material). A score of zero indicates no academic gender bias. **(B)** Mean explicit science = male score (+/- 1 *se*) by sex and major field. Majors are ordered, left to right, by ratings of science content (method described in Supplementary Material). A score of zero indicates no academic gender bias. **(C)** Mean explicit arts = female score (+/- 1 *se*) by sex and major field. Majors are ordered, left to right, by ratings of science content (method described in Supplementary Material). A score of zero indicates no academic gender bias.

Following other researchers in the STEM achievement literature (e.g., Elliott et al., 1995; Xie and Shauman, 2003; Smyth and McArdle, 2004; Tai et al., 2006), we defined STEM majors as those in biological, physical, computer, or health^2^ sciences (those choosing the “Other” option, about 6% of respondents, were excluded from analyses). For the purpose of displaying the dozen categories of majors in Figure 2A through Figure 4B, from least to most science-intensive, we asked 19 psychology graduate students who were blind to our hypotheses and analytic plan to rank the categories in order of their perception of the amount of scientific course work required (Cronbach's α = 0.985; see Supplementary Materials for details).

**Figure 3:**
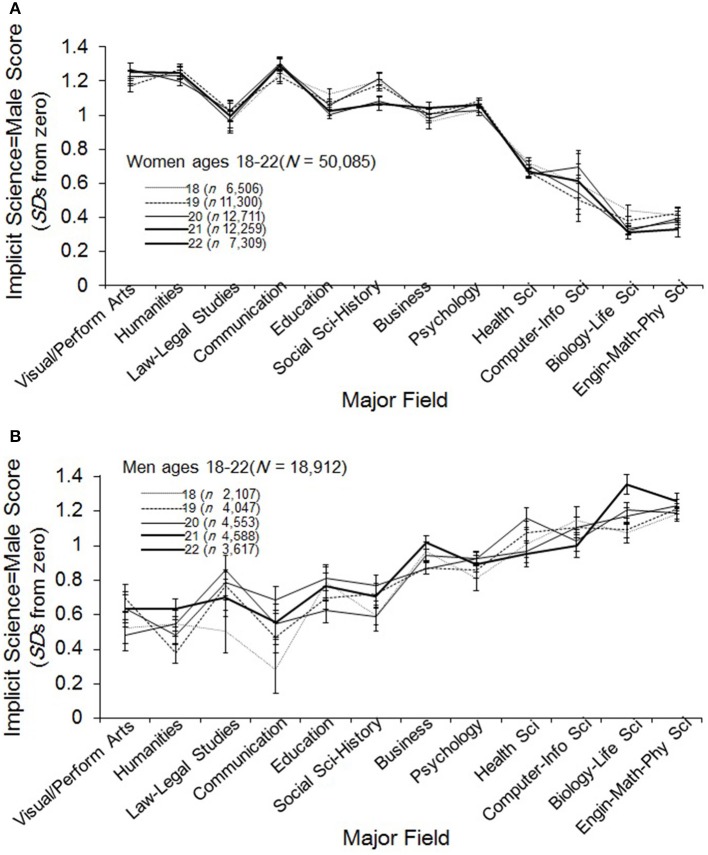
**(A)** Mean implicit science = male IAT score (+/- 1 *se*) for women ages 18–22 by age and major field. Majors are ordered, left to right, by ratings of science content (method described in Supplementary Material). A score of zero indicates no academic gender bias. **(B)** Mean implicit science = male score (+/- 1 *se*) for men ages 18–22 by age and major field. Majors are ordered, left to right, by ratings of science content (method described in Supplementary Material). A score of zero indicates no academic gender bias.

**Figure 4:**
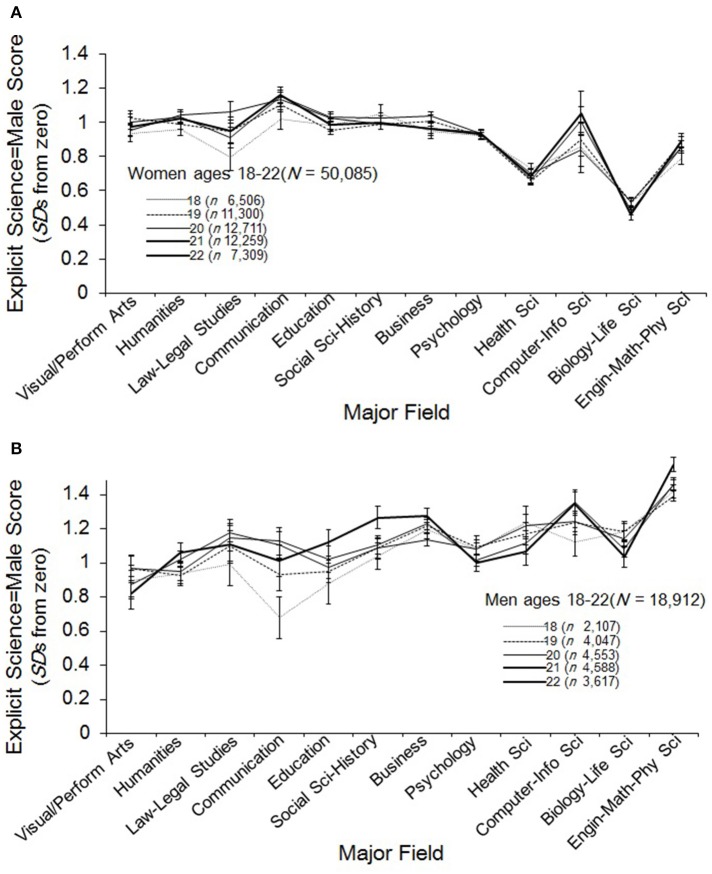
**(A)** Mean explicit science = male score (+/- 1 *se*) for women ages 18–22 by age and major field. Majors are ordered, left to right, by ratings of science content (method described in Supplementary Material). A score of zero indicates no academic gender bias. **(B)** Mean explicit science = male score (+/- 1 *se*) for men ages 18–22 by age and major field. Majors are ordered, left to right, by ratings of science content (method described in Supplementary Material). A score of zero indicates no academic gender bias.

### Explicit science identity: scientific profession

A question about occupation was added to the *Project Implicit* survey in December 2006. Our analyses focus on comparisons between respondents who identified, by both occupation and corresponding education level, as engineers, physicians, biological scientists, or physical scientists and reported an age of 26 or older (*N* = 4593, which constituted 12% of occupations reported by participants in that age range). Age 26 was used as a threshold to roughly control for the youngest typical age of attaining an MD degree in the U.S. (Association of American Medical Colleges, 2014).

### Explicit science identity: importance of being personally knowledgeable about science

Also added to the survey in December 2006 were questions about personal knowledge goals in three broad domains, liberal arts, math, and science. Specifically, each participant was asked about a random two of the three, as follows: “*Rate the following personal-goal-statements on their importance to you”*:

“*Being knowledgeable about liberal arts.” “… about math.” “… about science.”*

The five rating options, with our coding in parentheses, were: Not at all important (0), Slightly (1), Moderately (2), Very (3), and Extremely important (4). The science question was answered by *N* = 69,929 participants.

### Mapping our four STEM academic major categories to collegiate and professional STEM gender ratios

Based on our review of NSF data (National Science Foundation, National Center for Science and Engineering Statistics, 2011a), we classified as either relatively high-female or low-female the gender ratios of the four STEM major categories from which our participants could choose:

High-female:
Biological sciences/life sciences.
Health professions or related sciences.
Low-female:
Computer and information sciences.
Engineering, mathematics, or physical sciences/science technologies.

The biological and health science fields are classified as high-female because women have recently constituted half or more of college graduates and employed scientists, while engineering, physical and computer science fields are classified as low-female because women tend to constitute less than one-third of undergraduates and scientists in these areas. While there is considerable variation of gender ratios within the disciplines of our category *Engineering, mathematics, or physical sciences/science technologies* (e.g., considering bachelor's degrees awarded in 2008, female proportions were 50% in chemistry, 44% in mathematics, 40% in earth sciences, 20% in physics, and 19% in engineering), nearly half of women earning degrees in these areas are in the particularly low-female ratio fields of engineering and physics (National Science Foundation, National Center for Science and Engineering Statistics, 2011a). If the proportions of women recently graduating in these subfields match the underlying proportions among our volunteers choosing the physical sciences category, then a reasonable approximation of the upper limit for the average percentage of women encountered by those reporting a physical science major is 32%.

### Explicit academic gender stereotypes

Explicit academic gender stereotypes were assessed separately for both “Liberal Arts” and “Science” by asking participants to “*Please rate how much you associate the following domains with males or females*.” Five response options were provided on the questionnaire until December of 2006 (strongly male, somewhat male, neither male nor female, somewhat female, strongly female) and seven options were provided thereafter (replacing the “somewhat” options with “moderately” and “slightly” options). Thus, 40% of participants answered with a 5-point scale and 60% with a 7-point scale. Regardless of scale type, a “neither male nor female” response was coded zero, stereotype-congruent responses were coded with positive integers and stereotype-incongruent responses were coded with negative integers (i.e., for the science–gender item, coding was either −2 to 2 or −3 to 3, with positive scores indicating stronger science–male associations; while for the arts–gender item, positive scores indicate stronger arts–female associations). To facilitate comparison of scores across the 5- and 7-point scales, scores were standardized within scale-type relative to a score of zero (means for 5- and 7-point standardized scores for science–male stereotype were 0.99 and 1.01, respectively, and 0.66 and 0.67, respectively, for the 5- and 7-point arts–female stereotype).

### Implicit academic gender stereotypes

The IAT assesses the relative strengths of cognitive associations and was administered according to the recommendations of Nosek et al. (2005). The gender–science IAT required quickly sorting words into one of four designated categories—*Female*, *Male*, *Liberal Arts*, or *Science—*using two computer keys. Training established the proper category for four corresponding sets of words: for example, Woman, Mother and Wife with “Female”; Man, Father and Husband with “Male”; Arts, Literature and Philosophy with “Liberal Arts”; and Biology, Chemistry and Physics with “Science” (complete list can be seen in the Supplementary Materials). Each participant sorted under two conditions: (1) stereotype-congruent, in which *science* and *male* words were sorted with one key, *liberal arts* and *female* words with the other; and (2) stereotype-incongruent, in which *science* and *female* words were sorted with one key, *liberal arts* and *male* words with the other. The order of the conditions was randomized. Faster correct sorting in the stereotype-congruent condition than in the stereotype-incongruent condition indicates greater strength of science–male (and liberal arts–female) associations relative to science–female (and liberal arts–male) associations. Data were cleaned according to guidelines recommended by Nosek et al. (2005) and used by Nosek et al. (2009) to guard against careless responding. These procedures resulted in disqualification of IAT data for 11% of respondents (see Supplementary Materials for details). An IAT *D* score was computed for each participant by taking the difference in mean response latency between the conditions and scaling it by the overall variation (*SD*) of the participant's response latencies (Greenwald et al., 2003). Raw *D* scores were then standardized for the entire sample relative to a score of zero, thus allowing standard-deviation-unit comparisons with the explicit stereotype scores. For the sake of simplicity we refer to this measure as the “gender–science” IAT and say, for example, that positive scores indicate science–male stereotyping. However, it is important to note that arts–female associations are an integral, inseparable component of this IAT (Nosek et al., 2005).

### Procedure

Upon entering the online *Project Implicit Demonstration* portal, participants were presented, in randomized order, with a list of topics from which to choose. Those who selected “Gender–Science,” were presented with three study components in randomized order: (1) a questionnaire about academic attitudes, goals and stereotypes, (2) the gender–science IAT, and (3) a brief demographic questionnaire.

## Results

Given the large sample sizes, even very small differences between means are significant at *p* < 0.0001. Therefore, we focus our reporting on the effect sizes, mostly Cohen's *d*s, and the reader can assume that if *p*-values are not given, they are less than 0.0001. Following Halpern et al.'s (2007) report on sex differences in science and math, we use the term *sex* when distinguishing men's and women's cognitions. To facilitate comparability, both implicit (*Istd*) and explicit (*Estd*) stereotype scores are expressed in standard deviation units relative to a zero, or no bias, score. There are two sets of analyses for each of our hypotheses, one with participants grouped by academic major, and another focused on those reporting scientific professions. Descriptive statistics are listed in Table 1 and all data and materials are available at the Open Science Framework (https://osf.io/y7a3n).

**Table 1:** **Descriptive statistics for stereotypes and importance of scientific knowledge by sex and academic major of highest degree**.

	**Gender stereotypes, *M* (*SD*)**					**Goal: science knowledge**		
	***N***	***N*-pct**	***I*-std**	***E*-Scistd**	***E*-Artstd**	***N***	***M* (*SD*)**	**Extreme%**
**WOMEN**								
Visual or performing arts	4348	3.5	1.23 (0.88)	0.98 (0.94)	0.55 (0.96)	1662	2.44 (1.01)	15
Humanities/liberal arts	12,522	10.1	1.28 (0.91)	1.07 (0.97)	0.71 (1.02)	4549	2.48 (0.97)	16
Law or legal studies	4694	3.8	0.99 (0.96)	0.98 (1.01)	0.70 (1.02)	1654	2.52 (0.98)	17
Communications	4495	3.6	1.28 (0.89)	1.12 (0.97)	0.89 (1.03)	1665	2.30 (0.99)	11
Education	16,808	13.5	1.05 (0.95)	1.00 (0.99)	0.80 (1.02)	6519	2.62 (0.95)	19
Social sciences or history	8760	7.0	1.15 (0.93)	1.03 (0.97)	0.72 (1.01)	3332	2.55 (0.95)	16
Business	12,520	10.1	0.99 (0.92)	1.03 (1.03)	0.81 (1.04)	4878	2.43 (0.98)	13
Psychology	20,547	16.5	1.05 (0.94)	0.95 (0.96)	0.82 (0.99)	8414	2.73 (0.92)	21
Health or related sciences	14,403	11.6	0.73 (0.98)	0.72 (1.01)	0.68 (0.99)	6337	3.22 (0.80)	42
Computer and info sciences	2607	2.1	0.74 (0.99)	0.98 (1.01)	0.65 (0.99)	919	3.01 (0.90)	34
Bio/life sciences	12,755	10.2	0.35 (1.02)	0.58 (0.95)	0.56 (0.91)	5558	3.61 (0.59)	66
Engin, math, phys sciences	10,020	8.0	0.39 (1.03)	0.88 (0.97)	0.61 (0.92)	4269	3.46 (0.71)	57
**All**	**124,479**		**0.92 (1.00)**	**0.92 (0.99)**	**0.72 (1.00)**	**49,756**	**2.85 (0.98)**	**30**
**MEN**								
Visual or performing arts	1954	3.7	0.65 (0.99)	0.98 (0.98)	0.19 (0.88)	635	2.59 (0.95)	17
Humanities/liberal arts	5254	10.0	0.64 (1.03)	1.06 (1.00)	0.24 (0.90)	1837	2.53 (0.97)	17
Law or legal studies	2440	4.7	0.80 (1.01)	1.17 (1.04)	0.45 (1.02)	976	2.56 (0.96)	17
Communications	1522	2.9	0.67 (1.00)	1.06 (1.02)	0.38 (0.97)	551	2.40 (0.99)	13
Education	3179	6.1	0.83 (1.00)	1.06 (0.99)	0.52 (1.00)	1254	2.67 (0.95)	20
Social sciences or history	3393	6.5	0.73 (1.00)	1.11 (0.99)	0.34 (0.97)	1251	2.65 (0.95	20
Business	7853	15.0	1.00 (0.95)	1.25 (1.00)	0.71 (1.07)	3102	2.57 (0.92)	15
Psychology	4779	9.1	0.91 (0.99)	1.08 (0.96)	0.50 (0.97)	1888	2.83 (0.92)	26
Health or related sciences	2899	5.5	1.12 (0.94)	1.14 (0.98)	0.63 (1.01)	1282	3.28 (0.77)	45
Computer and info sciences	4257	8.1	1.09 (0.94)	1.32 (0.99)	0.62 (0.99)	1480	3.09 (0.81)	33
Bio/life sciences	4433	8.5	1.18 (0.95)	1.07 (0.95)	0.56 (0.96)	1834	3.53 (0.63)	60
Engin, math, phys sciences	10,493	20.0	1.21 (0.96)	1.39 (0.96)	0.64 (0.97)	4083	3.39 (0.73)	51
**All**	**52,456**		**0.97 (0.99)**	**1.19 (0.99)**	**0.53 (0.99)**	**20,173**	**2.93 (0.94)**	**32**

*Categories of majors are ordered, top to bottom, from lowest to highest science-content ratings made by psychology graduate students blind to our study hypotheses. Stereotype scores (suffix std) are standardized across the full sample relative to a score of zero. Stereotype labels: I, Implicit science-male; E-Sci, Explicit science-male; E-Art, Explicit arts-female. Response scale for the “Goal: Science Knowledge” variable was Not at all important, Slightly important, Moderately important, Very important, and Extremely important, and is coded 0–4. Extreme% indicates the percentage of respondents choosing Extremely important*.

## Hypothesis 1a: implicit stereotype differences between female and male scientists

*Women who are strongly identified with science will have relatively weak implicit stereotypes, while men who are strongly science-identified will have relatively strong ones*.

### Identification by academic major

Averaged across the entire sample, implicit science–male stereotyping was strong, nearly a full standard deviation above zero, *Istd* = 0.93. Overall, participant sex made a trivial difference, men averaging higher by just 0.05 standard deviations (i.e., Cohen's *d* = 0.05). However, as predicted, substantial sex differences were observed when participants were grouped by their academic major (Figure 2A), the direction of the difference varying systematically with rankings of the majors' degree of science content. The largest differences between men and women (*d*s ~ 0.8) came in the fields rated highest in scientific content (biological and physical sciences), where men's stereotyping was the strongest among all men and women's was the weakest among all women. This pattern conforms to Greenwald et al.'s (2002) cognitive consistency principles. That is, the strongest science–male (liberal arts–female) stereotypes are observed among those whose sex is aligned with their major in a stereotype-congruent fashion (e.g., among women identified with strongly non-STEM majors like arts and humanities, and among men identified with STEM majors), while the weakest stereotypes are seen among those with stereotype-incongruent combinations (men in arts and women in STEM). This pattern makes clear that this implicit stereotype is not simply a socially-shared association acquired through cultural exposure. Rather, it reveals important dependencies with combinations of gender identity and science/arts identities.

While women in STEM have the weakest science–male stereotypes, suggesting an important relation with their scientific identity, it is notable that they do not evidence a counter-stereotypical implicit association. On average, women majoring in STEM of any kind (the four right-most groups of majors in Figure 2A) still implicitly stereotyped science as male by half a standard deviation above the zero-point of no stereotyping (*Istd* = 0.53), and even those in the two categories of majors with the lowest average stereotypes, biological and physical sciences, still evidenced stereotypes of at least a third of a standard deviation in the science-is-male direction, *Istd* 0.33 and 0.39, respectively.

### Identification by scientific profession

**Figure 5:**
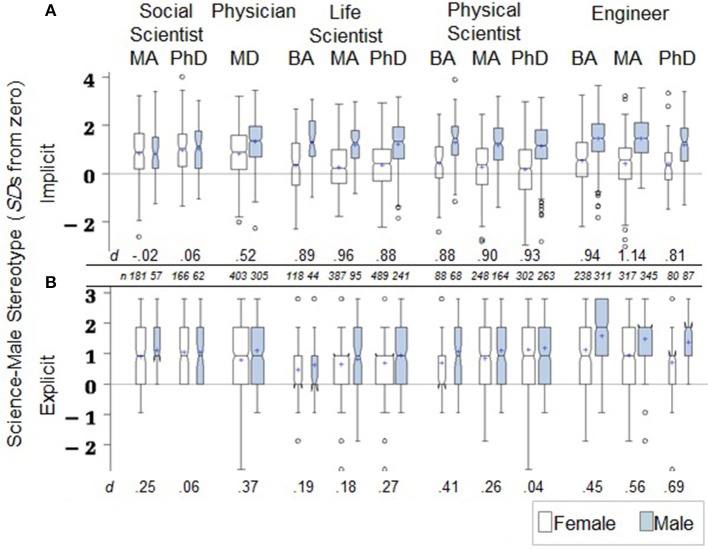
**(A,B)** Boxplots of stereotype scores by sex and science profession for participants age 26 and older. Box width is proportional to subgroup *n* and notches indicate standard errors around the median. *d* scores are standardized sex effects, male mean minus female mean, within each profession/degree category.

When participants are classified by scientific profession (see Figure 5A), we find the same general sex-effect pattern that was observed within academic major classifications—stronger stereotyping by men than women (this is not the case among social scientists, shown only for comparison, who stereotyped at a robust level but without sex differences). Male physicians, life scientists, physical scientists, and engineers all evidence much stronger levels of implicit science–male stereotype than the women in these professions, with a median sex-difference effect size of *d* of 0.89. Indeed, the smallest *d*, 0.52 for the physicians, is something of an outlier, with the next smallest effect being *d* = 0.81. The smaller sex effect for the physicians is driven by the relatively stronger average stereotype evidenced by the women, which is higher than that of the next highest female group in Figure 5A—engineers with bachelor's degrees—by a *d* of 0.29.

## Hypothesis 1b: explicit stereotype differences between female and male scientists

*Women who are strongly identified with science will have relatively weak explicit science-is-male stereotypes, while men who are strongly science-identified will have relatively strong ones. Conscious motivations to respond in accord with both personally- and societally-endorsed values, however, are expected to constrain the magnitude of effects relative to those for the implicit stereotype*.

### Identification by academic major

Responses to the two explicit stereotype measures, science–gender and arts–gender, are shown as a function of major in Figures 2B,C. As was the case for implicit stereotypes, overall means averaged strongly in stereotypical directions (science–male and arts–female), though associations of science with male were stronger than those of arts with female, *Estd* = 1.01 and 0.66, respectively. Sixty-three percent of participants reported associating science at least “slightly” with male, compared with 46% who associated arts at least “slightly” with female. Science–male and arts–female ratings were not highly correlated (*r* = 0.28).

Unlike for implicit stereotyping, there were overall sex differences in explicit stereotyping, with men more likely than women to associate science with male (70 vs. 60%), and women more likely than men to associate arts with female (49 vs. 41%). As noticeable in Figures 2B,C, these sex differences are driven by participants in the corresponding STEM and non-STEM majors, respectively. That is, the sex difference in science stereotyping is primarily among STEM majors and the sex difference in arts stereotyping is among non-STEM majors. Supporting our hypothesis, the sex differences in explicit science stereotyping were largest among the STEM majors, men stereotyping more strongly than women (*d*s ranging 0.35–0.52), but smaller than the sex differences observed for implicit stereotyping. The sex difference in arts stereotyping, however (Figure 2C), owed virtually nothing to STEM majors (a *d* of 0.06 is the largest sex difference in any of the STEM fields). It came primarily, instead, from those in the eight *non*-STEM fields (median *d* = 0.35), women stereotyping more than men.

### Identification by scientific profession

The same, expected pattern of stronger stereotyping by men in STEM was also observed for the explicit gender stereotypes of science professionals aged 26 and older (see Figure 5B). Male physicians, biological and physical scientists, and engineers (*N* = 1923) averaged *Estd* = 1.23, *SD* = 0.99, while women in these fields (*N* = 2670) averaged *Estd* = 0.82, *SD* = 0.98, for a *d* = 0.41. The magnitude of the sex difference in explicit stereotyping is, again, less than for implicit stereotyping.

## Hypothesis 2a: implicit stereotype differences as a function of gender ratios in science environments

*Implicit science-is-male stereotypes will be stronger for both women and men in low-female STEM fields than in high-female fields, though sex differences should remain*.

### Identification by academic major

The primary question of our study is whether science-is-male stereotypes vary as a function of gender ratio differences across scientific disciplines. First, it is apparent from Figure 2A that variation in average implicit stereotyping across the four science domains (at right) is greater for women than for men. Within-sex analysis of variance (ANOVA) modeling for participants in these domains yielded an extremely small effect of scientific category for men, *R*^2^ = 0.003, *F*_(3, 22078)_ = 20.9 (*Istd* = 1.17, *SD* = 0.95), but a larger one for women, *R*^2^ = 0.031, *F*_(3, 39781)_ = 421.6 (*Istd* = 0.52, *SD* = 1.02). The noticeable stereotyping difference for women does not align with differences in gender ratios across the scientific disciplines. If stereotypes covaried with gender ratios, we would expect differences in the strength of stereotypes evidenced by women in the health vs. computer science fields (which are high- and low-female, respectively) and in biological vs. physical sciences (also high- and low-female). We find, however, that the stereotype strengths for each of these comparisons differ very little, *F*_(1, 17008)_ = 0.2, *p* = 0.68, for health vs. computer science, and *F*_(1, 22773)_ = 7.4, *p* = 0.007, *d* = 0.037, for biological vs. physical sciences. The noticeable difference falls, instead, between health and computer sciences combined, on one hand (average *Istd* = 0.73, *SD* = 0.98), and biological and physical sciences combined on the other (average *Istd* = 0.37, *SD* = 1.03). The implicit stereotyping difference between these two combinations was more than a third of a standard deviation, *d* = 0.37, *R*^2^ = 0.031, *F*_(1, 39783)_ = 1257.

These patterns do not support our hypothesis that strength of implicit stereotyping among those in science domains will vary with gender ratios. Rather, the noticeable difference for women tracked with differences in scientific values as indicated by their ratings of the personal importance of scientific knowledge. Those with the weakest stereotypes—the women in biological and physical sciences—assigned the greatest importance to the personal goal of being knowledgeable in science. As seen in Table 1, 66% of biological sciences women and 57% of physical sciences women rated knowledge of science as an extremely important personal goal, compared with only 42 and 34%, respectively, of women with health and computer sciences majors. Following Baron and Kenny's recommended steps (Baron and Kenny, 1986; Kenny, 2014), we used three regression models to evaluate the science-knowledge-importance variable as a potential mediator of the difference in implicit stereotyping between biological/physical sciences women and health/computer sciences women. Model 1 estimated the bivariate regression between group membership (X, dummy-coded 0 for health/computer science majors and 1 for biological/physical science majors) and implicit stereotyping (Y); Model 2 estimated the bivariate regression between X and the proposed mediator (M, the science-knowledge-importance variable, coded 0–4); and Model 3 estimated the multiple regression of implicit stereotyping on both X and M. The baseline effect (Model 1) of X (being a biological/physical science major) on implicit stereotyping was estimated as *b* = −0.36, model *R*^2^ = 0.031. Model 2 demonstrated that type of science field (X) predicts science-knowledge-importance score (M), *b* = 0.35, model *R*^2^ = 0.054. When both X and M were included in the multiple regression Model 3, the effect of being a biological/physical science major was reduced to *b* = −0.29, a reduction of about one-fifth compared to the estimate from Model 1, and model *R*^2^ nearly doubled to 0.057. Thus, an indicator of the strength of personal scientific values provided some traction in accounting for stereotyping differences among women in the different science groups.

### Identification by scientific profession

Since the sex effects (Hypothesis 1a; Figure 5A) were fairly uniformly large and not our critical question, we fit separate within-sex bivariate regression models to estimate gender-ratio effects. First, as previously noted, female physicians had stronger implicit stereotypes than women in any other STEM professional group. This is incongruent with the gender-ratios hypothesis that predicts weaker stereotyping among physicians, relative to physical scientists or engineers, given the relatively high-female ratio in medicine.

Among the remaining types of scientists, we estimated gender-ratio effects by contrasting the implicit stereotypes of life scientists (coded 0), where high-female ratios are more likely, with those of physical scientists and engineers (coded 1), where low-female ratios are more likely. Results of regression analyses predicting implicit stereotyping from this contrast of disciplines were non-significant for both women and men (for women, *b* = 0.000, *t* = −0.01, *p* = 0.99, *R*^2^ = 0.000; for men, *b* = 0.135, *t* = 2.67, *p* = 0.008, *R*^2^ = 0.004). Thus, our gender-ratio hypothesis for implicit stereotyping is not supported when tested among professional scientists.

## Hypothesis 2b: explicit stereotype differences as a function of gender ratios in science environments

*Explicit science-is-male stereotypes will be stronger for both women and men in low-female STEM fields than in high-female fields, though, again, group variation on explicit stereotype means should be somewhat constrained by conscious values and motivations to respond without bias*.

### Identification by academic major

Unlike for implicit stereotyping, patterns of explicit science–male stereotyping conformed to our gender-ratio hypothesis (see Figure 2B). For both men and women in sciences, the weakest explicit stereotypes were in the domains where women are more strongly represented, i.e., in health and biological sciences, and the strongest were seen where women are least represented, in computer and physical sciences. Notably, for scientific men who are in high-female fields (health and biological sciences) stereotype levels are rather generic, i.e., similar to those among the non-STEM men and women. For such men, while their identity (“I'm scientific and I'm male”) maps onto the stereotype, their environment, on average, belies the stereotype (not clearly male-majority). It is only the men in majority-male environments, computer and physical sciences, who deviate (upward) from the generic level of stereotyping. For scientific women, the generic level is seen for those in the low-female fields, where, again, there is mismatch between their identity and the stereotype manifested in gender-ratios. In their case, however, personal identities, scientific and female, belie the stereotype and the environment supports it. The women whose stereotypes deviate (downward) from the generic tend to be in the high-female fields in which gender ratios complement their identities in undermining the stereotypical propositions they may consider when explicitly reporting gender–science associations.

### Identification by scientific profession

The explicit stereotypes of scientific professionals were also congruent with the gender-ratio predictions of hypothesis 2b. Physical scientists and engineers, together, had stronger stereotypes than life scientists, *d* effect sizes of 0.37 for women and 0.52 for men (the estimated stereotyping effect of being a physical scientist or engineer was *b* = 0.35, *t* = 9.64, *p* < 0.0001, *R*^2^ = 0.032 for women; *b* = 0.50, *t* = 9.81, *p* < 0.0001, *R*^2^ = 0.047, for men).

Since physicians' explicit science–male stereotypes did not obviously differ from those of the other STEM scientists, as female physicians' did for implicit stereotype, we included physicians in another set of regression analyses with PhD-level participants in the other STEM domains. That is, we contrasted physicians/life scientists at the MD or PhD-level (coded 0) with physical scientists/engineers at the PhD-level (coded 1). We relaxed alpha to 0.01 because of the smaller cell sizes (e.g., *N* = 350 male physical scientists/engineers with PhDs). Effects again supported our gender-ratio hypotheses, albeit less strongly among these MD/PhDs (for women, *b* = 0.30, *t* = 5.26, *p* < 0.0001, *R*^2^ = 0.022; for men, *b* = 0.20, *t* = 3.09, *p* = 0.0021, *R*^2^ = 0.011).

Thus, unlike results for the implicit stereotype, the patterns of explicit science-is-male stereotypes generally conform to our gender ratios hypothesis (2b) for STEM professionals. Physicians and life scientists, who are more likely to work in high-female ratio environments, explicitly stereotype science as male less strongly than do engineers and physical scientists, who are more likely to find themselves in low-female ratio work settings.

## Hypothesis 3a: implicit stereotype differences as a function of “dosage” of exposure to given gender-ratios

*Implicit science-is-male stereotyping should increase with prolonged exposure to low-female STEM environments and decrease with ongoing exposure to high-female STEM environments*.

### Identification by academic major

Women's means are plotted in Figure 3A for each year of age, 18–22, across all 12 academic categories. If length of exposure to collegiate science environments with skewed gender ratios has an effect on implicit stereotypes, then in the low-female computer and physical science domains we should see stereotype-strengthening across these ages, and weakening in the high-female health and biological science domains. We tested these expectations with ANOVAs contrasting stereotype means across the five age groups for each sex within each of our four categories of STEM majors. Given the smaller cell sizes in these models for the effect of age within sex-by-major groups (the smallest being the samples for women in computer science, ranging from *N* = 30–74), alpha for significance testing was reduced to 0.01.

Each of the ANOVA models for women was non-significant, bearing out the impression of stability suggested by the overlapping standard error bars around these means in Figure 3A^3^. Thus, for women in STEM fields, there was not statistically significant variation in implicit science stereotyping across groups spanning the traditional age range of college study.

For men, age effects were non-significant for all but one domain, biological science, *R*^2^ = 0.02, *F*_(4, 1152)_ = 6.23, *p* < 0.0001 (*Istd* = 1.23, *SD* = 0.90). The pattern among the men in biology was of increasing stereotype strength (see Figure 3B), despite the majority proportion of women among biology majors nationally. Eighteen-year-old men in biology averaged *Istd* = 1.13 (*SD* = 0.89), compared with *Istd* = 1.48 (*SD* = 0.84) for the 22-year-olds, an effect of *d* = 0.31. Thus, a lack of difference across years of age was the dominant finding for women and men, and the one instance of significant age effect is in a direction opposite to the gender-ratio dosage hypothesis.

### Identification by scientific profession

To test whether implicit stereotyping varies with increasing duration and intensity of training, indexed by degree level, we estimated regression models using scientist-type (life scientist, coded 0, vs. physical scientist/engineer, coded 1) and degree-level (bachelor's, master's, or PhD) as predictors. Alpha was set at 0.05 because of the relatively small numbers in some categories, e.g., *N* = 68 for male physical scientists with a bachelor's degree. Two orthogonal contrast codes were used to index degree-level effects, code-1 for Masters vs. PhD and code-2 for Bachelors vs. Masters and PhD, together. Because degree-level is confounded with age, age was included as a covariate in all models (and yielded a positive main effect, but no interactions, for both sexes).

No effects of degree-level were found for men, but for women a significant interaction was observed between scientist-type and Masters- vs. PhD-level degrees, *t* = −2.58, *p* = 0.01. Implicit stereotyping strengthened with higher degrees among the life scientist women, but weakened with higher degrees among physical scientists and engineers, a pattern precisely opposite to our hypothesis (3a) that greater tenure in a field would correlate with stronger or weaker implicit stereotype depending on female-male ratios. Specifically, among female life scientists, implicit stereotypes were *Istd* = 0.25 (*SD* = 0.97) at the Masters level and *Istd* = 0.37 (*SD* = 0.99) at the PhD level, compared with those of female physical scientists and engineers that were *Istd* = 0.31 (*SD* = 1.02) at the Masters level and *Istd* = 0.19 (*SD* = 1.05) at the PhD level. We hypothesized the opposite, that weaker stereotyping would occur with higher degree-attainment in the relatively high-female life sciences, and stronger stereotyping would occur with higher degree-level in the low-female physical and engineering sciences.

## Hypothesis 3b: explicit stereotype differences as a function of “dosage” of exposure to given gender-ratios

*Explicit stereotypes, when measured for scientists in a given field with roughly constant gender-ratio, will not be systematically responsive to dosage because the general propositions being weighed may not change (systematically) very much*.

### Identification by academic major

Figures 4A,B show plots for women and men, respectively, of explicit science–male stereotype means for each age across the academic major categories. The only instance of age differences in stereotyping at the 0.01 alpha level was a very small effect for men in physical sciences, *R*^2^ = 0.007, *F*_(4, 2211)_ = 3.76, *p* = 0.005 (*Estd* = 1.46, *SD* = 0.90). The peculiarity of this finding, the one significant test out of eight, warrants circumspection. Overall, the lack of variation in explicit stereotyping among STEM majors across the college years, supports our hypothesis of no systematic change for either men or women when the gender ratio of the given field is assumed constant.

### Identification by scientific profession

Using the same regression estimation approach as was described for the implicit stereotype analysis (contrast-coded predictors of scientist-type, life scientist vs. physical scientist/engineer, and degree-level, alpha 0.05), we found no dosage effect of degree level for explicit science–male stereotype among women, and a significant, but unpredicted interactive pattern for men similar to that observed for the implicit stereotyping of women, *t* = −2.11, *p* = 0.035. Among male life scientists, explicit stereotypes were stronger at the PhD level (*Istd* = 0.95, *SD* = 0.94) than at the Masters level (*Istd* = 0.75, *SD* = 0.95), but the opposite held for male physical scientists and engineers, who were weaker at the PhD level (*Istd* = 1.25, *SD* = 1.00) than at the Masters level (*Istd* = 1.41, *SD* = 0.96). This pattern supports our hypothesis that evidence of systematic change of explicit stereotyping was not expected within environments of particular gender ratios.

## Discussion

With a sample of 176,935, including thousands of engineers, physicians and scientists, we examined science-is-male stereotypic associations as a function of sex, scientific identity, and gender ratios in scientific disciplines. Stereotyping science as male was normative, implicitly and explicitly, as both types of scores averaged roughly a standard deviation above the zero-level of stereotyping on the respective scales. However, both types were marked by considerable variation depending on sex and academic or career identity, demonstrating that these gender associations are not simple reflections of a common cultural stereotype in the air. As expected, consistent with a well-established literature, we observed a positive relationship between stereotyping and science identity for men and a negative relationship for women. Men in STEM evidenced stronger science-is-male stereotypes than their non-STEM brethren, especially implicitly, while women in STEM evidenced the opposite pattern, much weaker implicit stereotyping than non-STEM women. As a result, in biological and physical sciences and engineering (the categories of science majors in our study that were rated as most scientific), the sex difference in implicit stereotyping was large, more than 0.8 standard deviations, ranking among the largest sex differences seen in cognitive research (Miller and Halpern, 2014).

Our primary question, however, was whether strength of science–male stereotyping would vary across scientific disciplines as a function of gender ratios in the disciplines. This hypothesis was supported for explicit stereotypes, but not for implicit ones. As expected, relatively stronger *explicit* stereotypes were evidenced by scientists studying and practicing in fields where women continue to be distinct minorities, and weaker ones were expressed by scientists in fields where women are better represented. *Implicit* stereotyping differences between scientists in different disciplines, however, did not correspond with gender ratios. For men there was little variation in implicit stereotype strength across four classifications of academic science concentration. Women, in contrast, evidenced considerable variation across these classifications, but it did not coincide with gender ratio differences. Rather, it coincided with differences in an indicator of the women's scientific identity. Specifically, implicit stereotyping varied with the value the women assigned to being personally knowledgeable about science. Women reporting that personal knowledge of science was “extremely important” had weaker implicit stereotypes than women reporting less personal priority on scientific knowledge. Though biological and physical science fields vary greatly in typical gender ratios, women in these disciplines were similar in the degree to which they placed extreme importance on personal scientific knowledge and in having the weakest implicit stereotypes of all women, whereas women in computer and health sciences, disciplines that also differ markedly in gender ratio, placed less importance on scientific knowledge and stereotyped more strongly. We found, furthermore, little evidence of difference in implicit stereotype strength corresponding to “dosage” of exposure to particular gender ratios. That is, within a particular field of whatever typical gender ratio, greater duration and intensity of exposure (whether operationalized by the cross-sectional proxy of traditional college ages from 18 to 22, or by levels of training among practicing scientists, BA, MA, or PhD) did not correspond to different implicit stereotype strengths as expected.

## Why didn't the implicit science–male stereotype vary with gender ratio differences in science fields?

While our analyses make clear that implicit gender–science stereotype strength varies greatly, primarily for women, across different scientific disciplines, gender ratios were not found to be a factor. How can this be if implicit stereotypes are sensitive to environmental inputs (Gawronski and Bodenhausen, 2006; Ratliff and Nosek, 2010; Miller et al., in press)? The answer may lie in Greenwald et al.'s (2002) assertion that the *self* is the power-center of automatic associative processes. Once strong self-concept bonds are formed (e.g., me-woman; me-science), the resulting, secondary, stereotypical associations (women-science) may be fairly impervious to local environmental conditions, like a preponderance of men in the lab, that would otherwise change them. Miller et al.'s (in press) country-level analysis identified precisely the relation between collegiate science gender-ratios and implicit stereotyping that we expected—but that we did not find—at a scientific discipline-level of analysis, i.e., higher female proportions in science associated with lower science-is-male stereotypes. We suspect that the apparent incongruence between their finding and ours hinges on scientific self-concept. That is, their analysis took into account respondents' country of citizenship and gender, but did not distinguish between levels of personal scientific identification, while ours controlled for self-reported academic major and priority on personal scientific knowledge. Our finding leads us to expect that the implicit stereotypes held by strongly science-identified women, like majors or scientists in biological or physical sciences, will be similar across countries, regardless of country proportion of women in collegiate science. That is, we would now expect science identity to trump the influence of local conditions.

Ratliff and Nosek (2010) note that, while implicit associative processes do a good job of accounting for covariation of events in the environment—like female proportions in science settings—they are also influenced by the frequency of association activations. Thus, if self-associations enjoy a leverage advantage in cognitive evaluative networks to begin with, as postulated by Greenwald et al. (2002), and self-associations also are more frequently activated than more abstract group-associations, then once a strong positive implicit science-self association is established (me-science), it may overpower potentially conflicting science-gender associations conveyed by the environment. We did not measure implicit science-self associations, but research indicates that they are strongly positively correlated with explicit indicators of science identity and favorability like ones we measured (Nosek and Smyth, 2011). Dasgupta's (2011) stereotype inoculation model hinges on developing a strong implicit STEM self-concept as a protection against pervasive cultural stereotypes and the vagaries of local conditions. Our data suggest that women in the most scientifically demanding fields, regardless of gender-ratios, are anchored at low levels of implicit stereotyping by their weighty scientific self-concepts and values.

Our “dosage” inference—that women's implicit stereotyping, within any particular academic major, is largely stable from age 18 on and across increasing levels of training and professional attainment—suggests that women's implicit stereotypes about gender and science may be fairly stable once strong scientific self-concepts are established. These cross-sectional data, however, can only be suggestive. Longitudinal research across the adult age range we studied is necessary for confidence in this pattern. Yet even if stability of adult implicit science associations was well-established, a more pressing longitudinal question would remain: How do children's and adolescents' implicit scientific associations develop and to what extent do they influence consequential STEM behaviors and choices? Galdi et al.'s (2014) demonstration that brief exposure to a stereotypical gender–math image vs. a counter-stereotypical one influenced both the implicit gender-math stereotyping and math performance of six-year-old girls should be a clarion call to such research. Longitudinal data on the development of implicit scientific self-concepts and stereotypes would help shed light on our “self-as-power-center” explanation for later stereotyping differences across scientific disciplines.

Based on their cross-sectional findings with elementary school students, that implicit math–gender stereotypes were already in force and were stronger than implicit math self-concepts, Cvencek et al. (2011) speculate that the implicit stereotype precedes, and may influence formation of, the implicit self-concept. It may be that stereotypes influence the early formation of self-concepts, but that once self-concepts are strong they are no longer easily influenced by stereotypes or stereotypical environmental conditions. Whatever the early trajectory and leading causal influences, Tai et al. (2006) found that by eighth grade, scientific goals—explicit values—were predictive of earning science degrees, especially in the physical sciences and engineering. It is time to add understanding of how implicit science stereotypes and self-concepts relate to such critical formative trajectories.

## Why did the explicit science–male stereotype vary with gender ratio differences in science fields?

According to Gawronski and Bodenhausen (2006), explicit associations are an amalgam of both automatic, associative processes, and controlled, propositional processes. The latter can be applied in deliberate attempts to adjust responses for the “truth-value” of evaluations or stereotypes. So in formulating their responses to the questionnaire item, “*Please rate how much you associate science with males or females*,” participants were able to exercise choice about how to weight possibly varying components of this association. A female physicist may have thought, for example, “Well, I love physics and am highly accomplished in the field, but I think this question is less about my personal experience and more about what I see as gender proportions in science, generally.” If most of her physics colleagues are male, such an interpretation of the question might have led her to select the “strongly male” gender-science association answer. Conversely, a female biology professor might have reasoned, “Most of my undergraduate students are female, and now a third of my faculty colleagues are female—with even higher proportions of women among the young stars—so I'll pick the ‘neither male nor female’ answer.” Though each of these hypothetical women would explicitly report a strong self-identification with science, their reports of gender associations with science can be made relatively independently of their self-concept. Our data suggest, however, that their implicit gender–science associations are a function of their self-associations with science, resulting in similarly weak implicit science–male stereotypes regardless of the different truth-values the gender ratios of their environments might have suggested.

## Limitations

Though the sample is large and there is more age and occupational variation than found in most studies of STEM stereotypes, it is not representative of any definable population. Participants are self-selected volunteers and their responses have no experimenter oversight. Generalizability to highly STEM-identified people is suggested, however, by our findings of comparable patterns of implicit stereotype strength and gender differences for University of Virginia undergraduates in engineering and advanced mathematics courses (Smyth unpublished manuscript; Martin et al., 2013; Smyth unpublished data). These students were not self-selected (their participation was a course requirement), yet the sex differences found in their stereotyping were similar in magnitude to those of the STEM-identified participants in the current study.

One reviewer expressed concern that the different methods of defining “Science” in our explicit and implicit stereotype instruments posed a potential confound for our results. Specifically, it was argued that the explicit stereotype instrument, asking participants to consider how strongly they associate males or females with the general concept, “Science,” presents an amorphous target that is likely to be interpreted through the lens of respondents' particular scientific discipline and experience, and so is prone to a correspondence between gender-ratios and the explicit stereotype. We agree with this interpretation and predicted that respondents' local experience would, indeed, inform their rating of the stereotype strength. “Science” in the *Implicit Association Test*, on the other hand, is ostensibly defined by all of the exemplars that are sorted into the category, including, for example, Biology and Chemistry, both relatively high-female fields, and Engineering and Physics, both low-female fields. Thus, the reviewer argues, the science construct used in the implicit measurement is more clearly defined as all-encompassing, and participants may be less likely to frame it in light of their particular disciplinary experience. We agree that this is possible, but believe that it is unlikely. A measurement property of the IAT is that the category labels dominate assessment over the exemplars (De Houwer, 2001; Nosek et al., 2007). The IAT in this study used the category labels “Science” and “Liberal Arts.” The individual exemplars may only have a small effect in as much as they change the construal of those category labels (Nosek et al., 2005). We think it is likely, therefore, that this implicit measure, like the explicit one, invokes a rather general science construct that is also subject to framing by respondents' particular experiences. Even so, replication with other explicit and implicit measurement techniques would be a useful check on this question.

Another impetus for replication with a different implicit science-is-male instrument is to avoid the IAT's structural requirement of a contrasting category, in this case, the fairly distinct other academic stereotype of “gender–arts.” Our explicit stereotype measurement approach, which allowed separate measurement of gender–science and gender–arts stereotypes, underscored their distinctiveness, especially for participants identifying as STEM majors (see Results for Hypothesis 1 and Figures 2B,C). Further evidence from our study suggests, however, that the science–gender construct may, indeed, be driving performance on this IAT more than the arts–gender construct. Among the STEM majors in our sample, explicit science–gender stereotype was a better predictor of IAT score than was the explicit arts–gender stereotype (regression *R*^2^ s of 0.063 and 0.013, respectively, and increasing only to 0.067 when both factors and their interaction were included in a multiple regression model).

Finally, our cross-sectional data require that inferences about the lack of environmental “dosage” effects be held cautiously until longitudinal studies are brought to bear. Of our two proxies for dosage (1) gradations of experience across the five years of traditional college age and (2) bachelors, masters and doctoral levels of scientific achievement, we agree with one of our reviewers that the latter is likely the more reliable. The dominant finding of scant evidence for dosage effects with either method, however, lends credence to the general conclusion that ongoing exposure to particular gender ratios, once strong scientific identities are established, may have little effect on personally-held stereotypes.

## Conclusion

Male scientists, on average, hold substantially stronger explicit and implicit science-is-male stereotypes than do female scientists. The gender difference is greatest, exceeding 0.8 standard deviations, for the implicit stereotypes held by men and women in either biological/life sciences or engineering/physical sciences (about twice the size of the differences in health and computer science fields). Average stereotype strengths differ across scientific disciplines, but in different patterns for explicit and implicit stereotypes. Differences in explicit stereotype strength correspond to gender ratios. That is, lower proportions of women in a field predict stronger explicit science-is-male associations. Implicit stereotype differences, in contrast, do not track with gender ratios. The implicit stereotyping levels for female and male scientists in life sciences, for example, where women are strongly represented, are similar to the levels in physical sciences and engineering, where women remain distinct minorities. Regardless of gender ratio, implicit stereotype differences align with indicators of individuals' scientific identity, such that disciplines with higher proportions of extremely science-identified people are characterized by more extreme implicit stereotype averages, extremely high for men and extremely low for women.

For scientifically-identified adults within a given discipline (assuming a generally constant gender-ratio), neither explicit nor implicit stereotype levels vary much as a function of cross-sectional proxies for “dosage” of the exposure to that gender ratio. That is, within disciplines, stereotype strengths are comparable between newly-declared STEM majors at age 18, bachelors, masters and PhD STEM degree-holders, and practicing scientists. Though stereotype change was not measured, these cross-sectional data suggest that, once a scientific identity is established, implicit stereotype strength remains fairly constant at a low level for women and at a high level for men, regardless of immediate gender ratio or duration and intensity of training and practice. They further suggest that neither sex differences in implicit stereotyping, nor individual differences in implicit stereotyping, are likely to account for women's differential representation across scientific disciplines *once a major is declared*.

This is not to suggest that adults' STEM interest and pursuit is not influenced by implicit stereotypes and self-concepts. There is much to learn, for instance, about implicit influences for the many who begin college without a clear major direction, as well as for the substantial number who intend a major in STEM at the start, but do not persist (Chang et al., 2014; Higher Education Research Institute, 2014). Still, it makes sense that research resources be focused at understanding the influences on children's self-concepts and stereotypes, as they are certainly more malleable and there is still time for interventions to work ahead of the coalescing of academic interests and goals during adolescence (Tai et al., 2006; Galdi et al., 2014). As noted by Ceci and Williams (2010), it is likely that a lion's share of the STEM sex difference derives from choices made prior to taking college courses.

Dasgupta (2011) emphasizes critical periods for inoculating girls' and women's implicit stereotype-incongruent self-concepts through increased exposure to same-sex peers and experts in the given domain. These critical inoculation periods are theorized to include youth, when self-concepts are forming, and times of academic or professional transition for adults, when decisions about persisting may be influenced unconsciously by feelings of belonging. Our finding that the implicit gender–science stereotypes of adults in science, while quite variable, do not depend on proportion of same-sex peers in the environment suggests that adults' implicit science self-concepts may also have little to do with gender ratios. That is, if Greenwald et al.'s (2002) balanced identity theory of implicit cognition is correct, the pattern of stability we have found for implicit science-is-male stereotypes should also hold for science self-concepts. Women in science, whether first-year collegians with a STEM major or PhD scientists, tend to have relatively weak implicit science-is-male stereotypes and can be expected to have strong implicit science self-concepts, regardless of gender proportions in the environment. Long-term longitudinal research is still lacking on these questions, but our results, combined with other evidence about critical junctures in the STEM pipeline, suggest that resources will likely be most fruitfully invested in studies beginning with children.

### Conflict of interest statement

Nosek is an officer and Smyth is a consultant of Project Implicit, Inc., a non-profit organization that includes in its mission “To develop and deliver methods for investigating and applying phenomena of implicit social cognition, including especially phenomena of implicit bias based on age, race, gender or other factors.”

## Footnotes

^1^Race and ethnicity proportions are based on participants since December 2006 (*N* = 106,515, or 60% of the sample) when new U.S. Office of Management and Budget reporting formats were adopted in the study. Racial/ethnic classification of earlier participants can be observed in the raw data available at https://osf.io/y7a3n/

^1^Note that some of our illustrative STEM statistics in the Introduction did not include Health sciences. Precise definitions of STEM vary, sometimes defined narrowly as natural science, technology, engineering, and mathematics fields, excluding Health and Social Sciences, and sometimes including all of these. Consistent with many researchers, our empirical analyses treat the health sciences as a STEM field.

^2^To insure that results were not affected by extreme observations, models were also run without the lowest and highest 1% of IAT scores within ageXgenderXmajor categories. ANOVA result patterns were unchanged under these conditions.
