# Supplementary material for: On the gender–science stereotypes held by scientists: explicit accord with gender-ratios, implicit accord with scientific identity
Source: Front Psychol. 2015 Apr 27;6:415. doi: 10.3389/fpsyg.2015.00415 (PMC4410517; doi:10.3389/fpsyg.2015.00415)
Supplement: Supplementary file 1 [file DataSheet1.DOCX]

*Supplementary Material*

On the Gender–Science Stereotypes held by Scientists:

Explicit accord with Gender-Ratios, Implicit accord with Scientific Identity

Frederick L. Smyth^1*^, Brian A. Nosek^1,2^

^1^Department of Psychology, University of Virginia, Charlottesville, VA, USA

^2^The Center for Open Science, Charlottesville, VA, USA

*** Correspondence:** Frederick L. Smyth, Department of Psychology, University of Virginia, Charlottesville, VA, USA, fsmyth@virginia.edu

Methodological Details

*Participant self-selection*

While very large, these data are not representative of U.S. citizens with at least some college education. Participants self-select when deciding to visit the Project Implicit website, in choosing the Gender–Science task (on the *Demonstration* site) and when persisting to completion. Choices might have been influenced by particular media reports, blog or chatroom discussions, recommendations of others, class or work assignments. The patterns of results evidenced by these highly self-selected participants have been replicated for less strongly self-selected ones, e.g., those who participate through the Project Implicit *Research* portal and are randomly assigned to the Gender–Science task, and University of Virginia undergraduates in advanced mathematics courses or engineering majors who participated as a course requirement.

*Effect of knowing the usual result?*

Upon entering the Project Implicit *Demonstration* portal, participants were presented, in randomized order, with a list of topics from which to choose, each including a brief description of the typical IAT result (in the case of the Gender–Science task, “*This IAT often reveals a relative link between liberal arts and females and between science and males*.”). Patterns of results for such participants do not differ from those of participants who were not alerted to the usual finding, e.g., those who participate through the Project Implicit *Research* portal are randomly assigned to the Gender–Science task and receive no forewarning about effects.

*Gender-Science IAT Categories and Stimuli*

**Male**

Boy

Man

Male

Father

Grandpa

Husband

Son

Uncle

**Female**

Girl

Woman

Female

Mother

Grandma

Wife

Daughter

Aunt

**Science** Astronomy

Biology

Chemistry

Engineering

Geology

Math

Physics

**Liberal Arts** Arts

English

History Humanities

Literature

Music

Philosophy

*IAT Cleaning and Scoring*

The IAT procedure followed the standard protocol described by Nosek et al. (2005) and was analyzed according to the improved *D* scoring algorithm (Greenwald et al., 2003): Responses faster than 400 milliseconds or slower than 10,000 milliseconds were removed, and errors were replaced with the mean of the correct responses in that response block plus a 600 millisecond penalty. Following Nosek et al. (2009), IAT scores were disqualified for any of the following criteria suggestive of careless participation: (1) going too fast (<300 ms) on more than 10% of the total test trials, (2) 25% of responses too fast in any one of the critical blocks, (3) 35% too fast in any one of the practice blocks, (4) making more than 30% erroneous responses across the critical blocks, (5) 40% errors in any one of the critical blocks, (6) 40% errors across all of the practice blocks, or (7) 50% errors in any one of the practice blocks. These standards resulted in a disqualification rate of 11%.

*Rating the Science-ness of Academic Major Categories*

For purposes of displaying in Figures 2-4 the twelve academic major categories in an approximate order by increasing science content, we asked 19 psychology graduate students who were unaware of our hypotheses to estimate the science requirements of majors in each category. Inter-rater reliability was high, Cronbach's α = .985. Instructions for raters were delivered via email as follows:

For a study examining how choices of college majors relate to gender stereotypes, I ask you to judge a list of college majors given below. Please place at the top of the list the major that you regard as requiring the greatest amount of scientific course work. At the bottom should be the major that you judge to require the least amount of scientific coursework.

Some categories lump together several majors that may differ in science requirements. For these do your best to make the requested judgment. If you cannot distinguish between categories, place them in a group (order within the group is unimportant). Put a blank line between the tied group and a more science-requiring major above and a less science-requiring major below.

If these instructions are unclear (they haven't been used before) please let me know. I will forward any clarifications to others.

The list is initially in alphabetical order. "There are no correct answers."

Please RESPOND ONLY TO ME so ratings will be independent. You can send back just the re-ordered list, with most scientific at top.

************************************ (Instructions) **************************************

Move to the top of this list the major subject that requires the greatest amount of scientific course work. At the bottom should be the major requiring the least amount of scientific coursework. Rank order those in between. When you cannot distinguish between majors, place them in a group (order within the group is unimportant). Put a blank line between the tied group of majors and a more science-requiring major above and a less science-requiring major below.

A. Biological sciences/life sciences

B. Business

C. Communications

D. Computer and information sciences

E. Education

F. Engineering, mathematics, or physical sciences/science technologies

G. Health professions or related sciences

H. Humanities/liberal arts

I. Law or legal studies

J. Psychology

K. Social sciences or history

L. Visual or performing arts

****************************************************************************************

*Explicit-Implicit Stereotype Relations*

The relation between scores on the gender–science–arts IAT and those on a composite of the explicit gender–science and gender–arts stereotypes (*r* = .21) was little stronger than that between the IAT and the explicit gender–science stereotype alone (*r* = .20). The IAT correlated *r* = .14 with the explicit gender–arts stereotype (scaled such that positive scores indicate arts–female associations). In multiple regression models predicting the implicit stereotype from the explicit stereotypes, the respective *R*^2^s for science stereotype alone, liberal arts alone, and the two together were .040, .020, and .048. Thus, explicit gender–science association uniquely accounted for 2.8% of the variation in implicit stereotyping, while explicit gender–arts association accounted uniquely for less than 1 percent. This pattern of superior predictive value for the science–male stereotype over the arts–female stereotype held regardless of participant sex and major (STEM or not). Thus, the implicit science–male/liberal arts–female stereotype, as measured by this IAT, is more related to explicit science–male associations than to explicit liberal arts–female ones—though not strongly related to either as is common with implicit–explicit relations for social attitudes and stereotypes (Gschwendner, Hofmann & Schmitt, 2008; Nosek, 2005).

*Notes on Figure 2a*

Deviations from the trend-lines in Figure 2a are noticeable for the categories of Law-Legal Studies and Education (weaker than expected stereotypes for women and stronger than expected for men), and likely reflect that some portion of such participants actually had an undergraduate substantive focus in STEM. The wording of our questions about academic major, “*Major field of study or that of your highest degree*,” did not allow for distinguishing undergraduate majors from graduate concentrations. For example, a law school graduate who majored in math as an undergraduate would properly answer that legal studies was the field of her or his highest degree and might select the physical sciences option as a second major.

References

Gschwendner, T., Hofmann, W., & Schmitt, M. (2008). Convergent and predictive validity of implicit and explicit anxiety measures as a function of specificity similarity and content similarity. *European Journal of Psychological Assessment, 24*, 254–262. doi:10.1027/1015-5759.24.4.254

Nosek, B. A. (2005). Moderators of the relationship between implicit and explicit evaluation. *Journal of Experimental Psychology: General, 134*, 565-584. doi:10.1037/0096-3445.134.4.565

Nosek, B. A., Greenwald, A. G., & Banaji, M. R. (2005). Understanding and using the Implicit Association Test: II. Method variables and construct validity. *Personality and Social Psychology Bulletin, 31*, 166-180. doi:10.1177/0146167204271418

Nosek, B. A., Smyth, F. L., Sriram, N., Lindner, N. M., Devos, T., Ayala, A. et al. (2009). National differences in gender-science stereotypes predict national sex differences in science and math achievement. *Proceedings of the National Academy of Sciences, 106*, 10593-10597. doi:10.1073/pnas.0809921106
